# Thermoregulatory response in juvenile *Hippocampus erectus*: Effect of magnitude and rate of thermal increase on metabolism and antioxidative defence

**DOI:** 10.1002/ece3.10977

**Published:** 2024-02-20

**Authors:** Giulia Del Vecchio, Gabriela Rodríguez‐Fuentes, Carlos Rosas, Maite Mascaró

**Affiliations:** ^1^ Posgrado en Ciencias del Mar y Limnología, Facultad de Ciencias Universidad Nacional Autónoma de México Sisal Yucatan Mexico; ^2^ Unidad de Química en Sisal, Facultad de Química Universidad Nacional Autónoma de México Sisal Yucatan Mexico; ^3^ Unidad Multidisciplinaria de Docencia e Investigación, Facultad de Ciencias Universidad Nacional Autónoma de México Sisal Yucatan Mexico

**Keywords:** antioxidative defence, ocean warming, routine metabolism, sea horses, thermal biology, thermal tolerance

## Abstract

Behavioural, physiological and biochemical mechanisms constitute the adaptive capacities that allow marine ectotherms to explore the environment beyond their thermal optimal. Limitations to the efficiency of these mechanisms define the transition from moderate to severe thermal stress, and serve to characterise the thermoregulatory response in the zone of thermal tolerance. We selected a tropical population of *Hippocampus erectus* to describe the timing of the physiological and biochemical mechanisms in response to the following increments in water temperature: (i) 4°C abrupt (26–30°C in <5 min); (ii) 7°C abrupt (26–33°C); (iii) 4°C gradual (1°C every 3 h) and (iv) 7°C gradual (1.5°C every 3 h). The routine metabolic rate (*Rrout*) of juvenile *H*. *erectus* was measured immediately before and after 0.5, 12 and 28 h of being exposed to each thermal treatment. Samples of muscle and abdominal organs were taken to quantify indicators of aerobic and anaerobic metabolism and antioxidant enzymes and oxidative stress at each moment throughout exposure. Results showed a full thermoregulatory response within 0.5 h: *Rrout* increased in direct correspondence with both the magnitude and rate of thermal increase; peroxidised lipids rapidly accumulated before the antioxidant defence was activated and early lactate concentrations suggested an immediate, yet temporary, reduction in aerobic scope. After 12 h, *Rrout* had decreased in sea horses exposed to 30°C, but not to 33°C, where *Rrout* continued high until the end of trials. Within 28 h of thermal exposure, all metabolite and antioxidant defence indicators had been restored to control levels (26°C). These findings testify to the outstanding thermal plasticity of *H*. *erectus* and explain their adjustment to rapid fluctuations in ambient temperature. Such features, however, do not protect this tropical population from the deleterious effects of chronic exposure to temperatures that have been predicted for the future.

## Graphical abstract

We selected a tropical population of *Hippocampus erectus* to describe the timing of the physiological and biochemical mechanisms in response to different magnitudes and rates thermal increase. A full thermoregulatory response was displayed within 0.5 h and all metabolite and antioxidant defence indicators had been restored to control levels (26°C) within 28 h of thermal exposure. These findings underline the sea horses' adaptive capacity to adjust to the rapid temporal fluctuations, but should not be interpreted as a robust shield against the deleterious effects of global warming on marine populations.

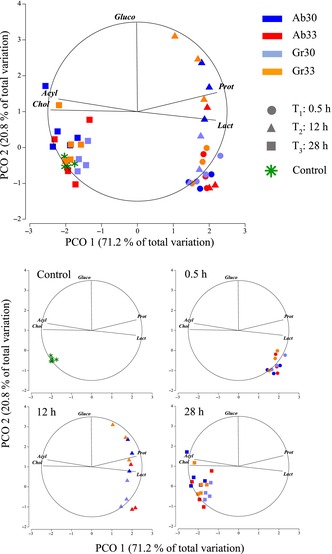

## 1 INTRODUCTION

By controlling the speed of enzymatic reactions, temperature modulates all metabolic processes in marine ectotherms (Roessig et al., 2004); it governs the physiological performance of individuals (Tepolt & Somero, 2014), thereby shaping the limits of the geographic distribution of their populations (Poloczanska et al., 2016; Wernberg et al., 2011).

The selective pressure of temperature on the physiology of marine ectotherms has resulted in adaptive capacities to inhabit environments characterised by marked thermal fluctuations (Pigliucci et al., 2006). These capacities are expressed through behavioural, physiological and biochemical mechanisms that operate for short periods, and allow individuals to search for food, refuge or reproduction sites beyond their thermal optimal (Farrell, 2016). The central goal of such mechanisms is to provide metabolic energy to satisfy basal maintenance (Pörtner et al., 2017) and protect cell structures and function from potentially hazardous disequilibrium (Rahaman & Rahaman, 2021; Regoli et al., 2011). Limitations to the amount of available energy and its rate of acquisition define the transition from moderate to severe thermal stress (Sokolova et al., 2012), thereby contributing to characterise the zone of thermal tolerance.

According to the oxygen‐ and capacity‐limited thermal tolerance hypothesis (OCLTT; Pörtner & Knust, 2007), the boundaries of the thermal optimal are known as *pejus* temperatures (*Tp* from Latin, meaning “to worsen”) and correspond to a reduction in the capacity to supply oxygen (hypoxaemia) hence the start of limitations in aerobic scope (i.e. the maximum amount of oxygen available for any aerobic activity above routine) as temperature increases or decreases. Beyond the permanently preferred temperature range, thermal tolerance is achieved by exploiting residual aerobic mechanisms of energy supply that will eventually be depleted (Pörtner et al., 2017). A critical thermal threshold (*Tc*) is reached when energy production turns mainly to anaerobic pathways (i.e. aerobic scope becomes minimal), and the mechanisms for the protection of cell membranes and structural proteins are activated (e.g. chaperones like heat shock proteins; Pörtner et al., 2017). At this stage, lactate accumulation and the production of reactive oxygen species (ROS) triggers enzymatic reactions of the antioxidant system aimed at restoring the redox balance in the cell cytoplasm (Giraud‐Billouda et al., 2019). If maintained, such thermal conditions lead to metabolic depression, and survival, thereof, will solely depend on time (Pörtner et al., 2017). The final thermal limit (*Td*) is related to the denaturation of proteins and has been situated beyond the Critical Thermal Maximum, defined as the extreme thermal threshold leading to the onset of spasms associated with unorganised locomotion (Leong et al., 2022).

The range of thermal tolerance of aquatic ectotherms is largely determined by the amplitude of temperature variation in their natural habitat across both temporal and spatial scales (Tepolt & Somero, 2014). This range is generally wider in temperate species than in those that inhabit colder or warmer, yet more stable water temperatures (Pörtner, 2002). The timing and magnitude of thermal fluctuations induce distinct organismal responses compared to those resulting from gradual directional change (e.g. climate change), or an abrupt environmental disruption (e.g. extreme weather events). There is substantial evidence indicating that extreme events have a greater influence on the performance and survival of ectotherms than the effects of gradually changing climates (Román‐Palacios & Wiens, 2020).

The lined sea horse, *Hippocampus erectus*, is an eurythermal species distributed along the Atlantic coast of the Americas from south of Nova Scotia Canada to the northern coast of Brazil (Lourie et al., 2016). Tropical populations of *H*. *erectus* are common in lagoons and wetlands characterised by marked environmental fluctuations over relatively short time scales. In Chelem, Yucatan, sea horses are exposed to annual thermal fluctuations from 19 to 31°C (Herrera‐Silveira & Morales‐Ojeda, 2010). The lagoon's shallow waters (0.25 and 1.5 m depth), however, result in marked daily and tidal variations in several abiotic conditions including temperature and dissolved oxygen (De la Lanza‐Espino & Gutiérrez‐Mendieta, 2017). During the warmest months of April and May, the mean temperature at the centre of the lagoon can vary between 22 and 30°C (Mariño‐Tapia & Enríquez, 2010), whereas shallow areas can register maximum temperatures of 38°C (CONANP, 2006).

Recent efforts investigating the thermal biology of *H*. *erectus* in Chelem have shown that sea horses tolerate abrupt and short‐lived changes in temperatures between 18 and 30°C, but their physiological performance decreases dangerously under scenarios of prolonged exposure to temperatures as common as 30°C (Huipe‐Zamora, 2015; Mascaró et al., 2016). Moreover, marked differences in physiological and behavioural indicators emerge when sea horses are exposed to gradual thermal increments in comparison to sustained sublethal temperatures (Mascaró et al., 2019). Response patterns similar to these have been observed in other species living in thermally variable environments, and have been explained as a greater capacity to increase metabolism that comes together with the limitations that result from the high metabolic costs associated (Magozzi & Calosi, 2014). The apparent paradox within this idea is relevant to studies on climate change since it has been suggested that species tolerant to extreme thermal events can be at a greater risk when exposed chronically to sub‐lethal temperatures (Vinagre et al., 2016).

The relation between the temporality and magnitude of thermal variation is complex and will ultimately affect the nature and scale of the physiological response of the organism (Kingsolver et al., 2015). Laboratory‐based experiments set to examine the thermal tolerance of aquatic species often describe the physiological conditions of organisms at the endpoint of an exposure to sublethal temperatures. However, the timing of the different biochemical and physiological mechanisms of adjustment throughout thermal exposure is rarely studied. Attention has been drawn to the importance of studies that integrate information on the time course of the thermal response in different levels of biological organisation (Somero, 2015). It is the aim of the present study to describe the physiological and biochemical mechanisms, as well as the moments and sequence in which they occur during the exposure of juvenile *H*. *erectus* to high temperatures in the limit of their thermal tolerance. This information will contribute to our understanding of the implications of thermal stress in terms of the energy balance and the capacity to restore homeostasis in this tropical population.

## 2 MATERIALS AND METHODS

This study followed the protocols for maintenance, manipulation and sacrifice of the experimental animals according to certified criteria established by the Institutional Committee for the Care and Use of Laboratory Animals (CICUAL) of the Faculty of Chemistry, UNAM (OFICIO/FQ/CICUAL/341/18). All efforts were made to minimise stress in experimental animals and meet standard levels of animal welfare (see below for conditions).

### 2.1 Origin and maintenance of experimental animals

Juvenile *H*. *erectus* used in the experiments came from four pregnant males collected at Laguna de Chelem, Yucatan, Mexico (21°17′N and 89°40′W) (SEMARNAT permit No. SGPA/DGVS/10959/15). Males were collected in February, when mean temperature and salinity at collection sites are 19.5 ± 0.5°C and 34.6 ± 1.1 psu respectively. Males were then transported to laboratory facilities at the Unidad Multidisciplinaria de Docencia e Investigación, Sisal, where they were individually placed in glass aquaria (30 cm width × 17 cm depth × 27 cm height; 14 L) with constant water salinity (35 psu) and temperature (26°C) until offspring was born.

New born sea horses were transferred to 10 L aquaria (7 or 8 individuals L^−1^) connected to a seawater recirculation system equipped with a mechanical (nylon monofilament bag) and a biological filter (with sand, coral fragments and artificial live rock fragments). Gentle aeration kept oxygen levels near saturation (~6 mg O_2_ L^−1^), and water conditions were maintained constant at 35 ± 1 ups and 26 ± 0.5°C to assure a similar thermal history for all experimental animals. Photoperiod was maintained at 10 h light:12 h dark with 1 h gradual changes between periods to resemble dusk and dawn. Braided raffia structures were introduced into each aquarium to serve as holdfast for the fish. Sea horses were fed nauplii and metanauplii of *Artemia salina* and frozen amphipods, *Paryhale hawaiensis*, following the maintenance protocols described in Mascaró et al. (2019).

### 2.2 Oxygen consumption

To examine the effect of the rate and magnitude of temperature increase on routine metabolic rate (*Rrout*, i.e., the minimum metabolic cost of maintaining biological functioning including energy expended on spontaneous movements to maintain posture; Chabot et al., 2016) of juvenile sea horses, the following thermal treatments were defined: (i) an abrupt increment (in <5 min) of 4°C from 26 to 30°C (Ab30); (ii) an abrupt increment (in <5 min) of 7°C from 26 to 33°C (Ab33); (iii) a gradual increment (1°C every 3 h) of 4°C from 26 to 30°C (Gr30) and (iv) a gradual increment (1.5°C every 3 h) of 7°C from 26 to 33°C (Gr33). Priority was given to attain the corresponding target temperatures in the same amount of time: 5 min and 12 h in abrupt and gradual change treatments respectively. Thus, the rates of thermal increase in Gr30 and Gr33 were different and valid comparisons were only made between Ab30 versus Gr30; Ab33 versus Gr33 and Ab30 versus Ab33.

Oxygen consumption rates were measured using flow‐through respirometry. Juveniles *H*. *erectus* were individually placed in cylindric respirometry chambers (250 mL) equipped with optic sensors (Loligo Systems) connected to an amplifier (OXY‐10 Mini Set PreSens©, Germany) that registered dissolved oxygen (mg L^−1^) every 15 s both at the entrance and exit of each chamber. Sensors were placed immediately at the water entrance and exit of the chamber, which in turn were separated by a distance of 2 cm. Sensors were calibrated with sea water at the corresponding target temperatures and 100% and 0% oxygen saturation using fully aerated sea water and a 5% anhydrous sodium sulphite solution. Chambers were connected to a recirculation system that maintained a water flux at 3.6 L h^−1^. An 80 L reservoir and a thermo‐regulator (TK1000, TECO, Italy) were used to control water temperature as required. The reservoir was equipped with a filtration system consisting of a cartridge (1 μm) and coral fragment and activated charcoal filters. Constant aeration within the reservoir guaranteed oxygen saturation levels at input in all treatment temperatures, whereas the flow‐through respirometry system maintained O_2_ concentrations within the chambers above 90% throughout measurements.

Twenty sea horses (*n* = 5 randomly assigned to each treatment) were placed in the respirometry chambers (at 26°C) for 15 h prior to thermal changes to habituate and remained 28 h after sea water had reached target temperatures in each case. To attain target temperatures in Ab30 and Ab33 water was exchanged directly in the reservoir, whereas the thermo‐regulator was used both to increase temperature in Gr30 and Gr33 and maintain it constant thereafter in all treatments. The five trials corresponding to each treatment were run simultaneously and one chamber was always left without a sea horse and used as a control; treatments were run one after another. Once trials ended, the wet weight (± 0.01 g) of each individual was registered (OHAUS Adventurer AR‐2140) and fish were euthanised by a cervical incision. The muscle tissue of the tail and abdominal organs of each individual was dissected on a cold plate (−4°C) and stored at −80°C in Eppendorf vials (1.5 mL) for further biochemical analyses.

Oxygen consumption was calculated using the following equation.

$${\mathrm{VO}}_{2}=\frac{\left(\left(\left[O_{2\;\mathrm{mg}\;L^{-1}}\right]\text{In}-\left[O_{2\;\mathrm{mg}\;L^{-1}}\right]\text{Out}\right)\cdot F\right)}{\mathrm{WW}}$$

where $\left[O_{2\;\mathrm{mg}\;L^{-1}}\right]\text{In}$ and $\left[O_{2\;\mathrm{mg}\;L^{-1}}\right]\text{Out}$ represent dissolved oxygen at the input and output of each chamber; F is the water flux in L h^−1^ and WW is the wet weight of each seahorse (g). The VO_2_ of the control chamber was subtracted from each individual value and the final rate was expressed in mg O_2_ g^−1^ h^−1^.

The mean value of individual VO_2_ every 20 min was obtained to build graphical representations of *Rrout* through time. These were then used to identify major changes in the patterns of *Rrout* and allowed to distinguish four relevant moments in relation to the thermal treatments applied: (i) 13 h prior to thermal change (T_0_); (ii) 0.5 h (T_1_); (iii) 12 h (T_2_) and (iv) 28 h (T3) measured after reaching the target temperature respectively.

Sea horses were approximately 6 months old and had a mean wet weight of 1.6 ± 0.4 g (*n* = 25) at the beginning of the experiment. Both male and female juveniles were indistinctly used to avoid excessive manipulation and error in sex identification. Sea horses remained unfed throughout trials (~50 h), but photoperiod was kept similar to that during maintenance to avoid the unwanted effect caused by variations in circadian rhythm.

### 2.3 Biochemical indicators of metabolism and antioxidant defence

Twenty‐four *H*. *erectus* (2.9 ± 1.2 g) were randomly and uniformly distributed in four glass aquaria (14 L) and exposed to the same thermal treatments. Three individuals from each treatment were sampled at 0.5 h (T_1_) and 12 h (T_2_) after reaching target temperatures in each case. Sampled fish were euthanised and stored as described previously, and added to complete a sampling design of at least three replicates in each combination of thermal treatment (Ab30, Ab33, Gr30, Gr33) and time (T_1_, T_2_, T_3_) and a control at constant 26°C for 28 h (C26). Sampling was designed to keep sacrificed animals to the minimum.

Samples of muscle tissue were used to quantify the following indicators of anaerobic and aerobic metabolism: lactate (Lact), glucose (Gluco), proteins (Prot), cholesterol (Chol) and acyl‐glycerides (Acyl). For metabolites quantification, 100–150 mg of tissue from each organism was subjected to five homogenisation cycles of 15 s each, with zirconium beads in buffer Tris pH 7.4. Samples were centrifuged (16,904 rcf, 4°C for 5 min) and the supernatant was then diluted with pyrogen‐free water in a ratio of 2:1 to the weight of the sample (final dilution factor 3×). Levels of metabolites were determined using commercial clinical diagnostic kits (ELITech Clinical Systems, France. Kits: CHSL‐0507 CHOLESTEROL PAP SL, GPSL‐0507 GLUCOSE PAP, TGML‐0427 TRIGLYCERIDES mono SL New and Trinity Biotech Lactate Reagent, Ireland) and values expressed in mg L^−1^.

Samples of the abdominal organs of sea horses (liver, stomach, intestine and reproductive organs) were used to quantify antioxidant enzymes and oxidative stress: total glutathione (GSH), superoxide dismutase (SOD), catalase (CAT), glutathione S‐transferase (GST), lipid peroxidation (LPO) and protein carbonylation (PO). Acetylcholinesterase (AChE) and carboxylesterase (CbE) were measured to assess esterase activity as a physiological condition factor. As described above, 100 mg of frozen sample were weighed and homogenised with 2 mL of Tris buffer pH 7.4. Special care was taken to keep the samples constantly cold to avoid degradation. Each sample's redox potential (mV) was measured with a potentiometer (Emyr, 6230NKA Jenco). Sigma Aldrich kits were used following the manufacturer's instructions to determine GST (kit CS04, based on Habig & Jakoby, 1981), total GSH (kit CA0260, based on Akerboom & Sies, 1981), SOD (kit 19160) and LPO (PeroxiDetect Kit). CAT was measured using the method described by Góth (1991) modified by Hadwan and Abed (2016). PO was measured following the protocol of Mesquita et al. (2014). Esterase activities were measured using methods described by Ellman et al. (1961) adapted by Rodríguez‐Fuentes et al. (2008) for AChE and by Hosokawa and Satoh (2002) for CbE.

### 2.4 Data analysis

Mean values of VO_2_ (± standard deviation) were calculated for *n* = 5 replicate measures in each treatment at T_0_, T_1_, T_2_, and T_3_ and converted to energy equivalents using an oxy‐caloric coefficient of 13.6 J mg^−1^ (Lucas & Watson, 2014). Because registers of VO_2_ through time lacked statistical independence, comparisons were limited to assess relative differences in the patterns of VO_2_ amongst treatments.

Temporal variations in the biochemical indicators of sea horse metabolism and antioxidant defence system were evaluated using principal coordinate analysis (PCoA). Three separate PCoA were obtained for metabolites (Lact, Gluco, Prot, Chol, Acyl), antioxidant enzymes and oxidative stress indicators (GSH, SOD, CAT, GST, LPO, PO) and esterase activity (AChE, CbE). Dissimilarity matrices of Euclidian distance between samples (Legendre & Legendre, 1998) were obtained once data had been transformed with either log or fourth root transformations (applied to metabolites concentration and enzyme activity respectively) and normalised. To facilitate the interpretation of PCoA, partial visualisations of treatments at different sampling moments were produced and presented in sequence together with the 2D configuration that included all samples combined.

A permutational multiple ANOVA was applied to each of the three multivariate data sets to detect statistical differences between centroids. The underlying model had thermal treatment (4 levels: Ab30, Ab33, Gr30, Gr33) and time (T_1_, T_2_, T_3_) as fixed factors and a hanging control (C26) with *n* = 3–5 replicates in each combination. Analyses comprised two stages: A first stage one‐way ANOVA with a = 13 levels of a single factor to contrast all treatment‐time combinations versus the control. If significant differences between control and no‐control conditions were detected, a second stage two‐way ANOVA with treatment (a = 4 levels) and time (b = 3 levels) as fixed factors was applied. In order to generate the empirical distributions of *pseudo‐F* values under the null hypotheses (Anderson, 2017), unrestricted permutations of raw data and permutations of residuals under the reduced model (9999) were used in the first and second stages respectively. Multivariate analysis and graphs were performed using PRIMER‐e v 7.0.17 PERMANOVA +1 ©.

## 3 RESULTS

No sea horses died during experimental trials indicating that a 28‐h exposure to either 30 or 33°C, attained either abruptly or gradually did not constitute an immediate risk for sea horse survival.

### 3.1 Oxygen consumption

**FIGURE 1:**
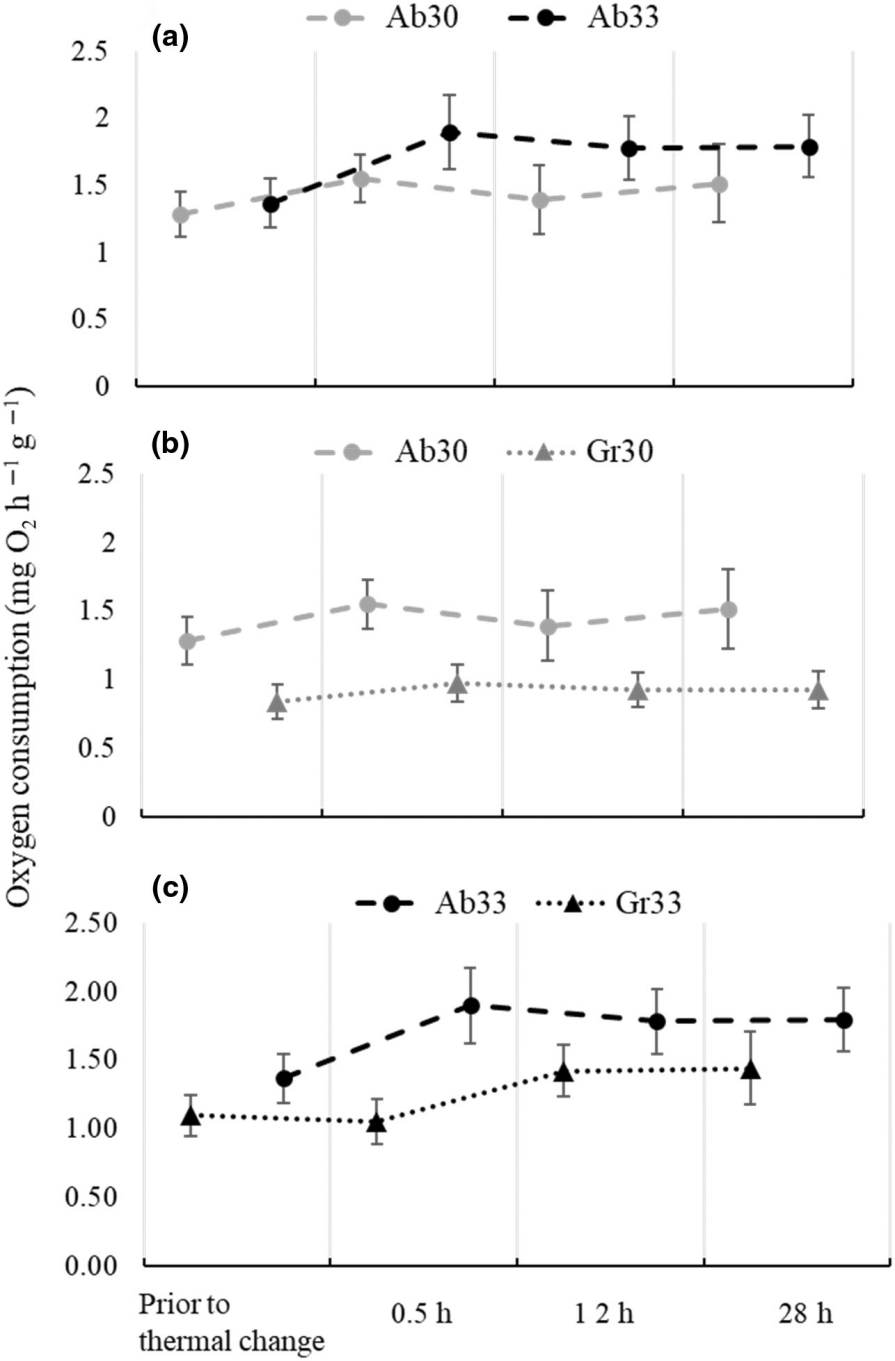
Oxygen consumption (mean values ± standard deviation) of juvenile *Hippocampus erectus* prior to thermal change and after 0.5, 12 and 28 h of exposure to (a) abrupt increments from 26 to 30°C (Ab 30) and 26 to 33°C (Ab 33); (b) abrupt (Ab 30) and gradual increments from 26 to 30°C (Gr 30) and (c) abrupt (Ab 33) and gradual increments from 26 to 33°C (Gr 33).

Oxygen consumption by juveniles of *H*. *erectus* prior to thermal change was markedly constant, with mean values of VO_2_ varying from 0.84 to 1.37 mgO_2_ h^−1^ g^−1^ in treatments Gr30 and Ab33 respectively (Table 1). In all treatments, sea horses immediately increased VO_2_ as a response to an abrupt thermal change, with a 39% increment after 0.5 h of exposure in Ab33 compared to only 20% increment in Ab30 (Table 1; Figure 1a). After 12 h of thermal exposure, juveniles in Ab30 had reduced VO_2_ to 1.39 ± 0.25 mgO_2_ h^−1^ g^−1^, scarcely 8% above the initial value. By contrast, sea horses in Ab33 maintained consumption at 1.78 ± 0.24 mgO_2_ h^−1^ g^−1^, still 30% above the initial VO_2_ value (Table 1; Figure 1a). After 28 h of exposure, sea horses in Ab30 had again increased VO_2_ to 1.52 ± 0.29, whereas those in Ab33 maintained a relatively high consumption of 1.79 ± 0.23 mgO_2_ h^−1^ g^−1^ (Table 1; Figure 1a).

**TABLE 1:** Oxygen consumption (VO_2_ mean values ± standard deviation) of juvenile *Hippocampus erectus* prior to thermal change and after 0.5, 12 and 28 h of exposure to abrupt and gradual increments from 26 to 30°C (4°C, Ab 30 and Gr 30) and 26 to 33°C (7°C, Ab 33 and Gr 33 respectively).

	Oxygen consumption (mg O_2_ h^−1^ g^−1^)							
	Ab 30	%	Ab 33	%	Gr 30	%	Gr 33	%
Prior to thermal change	1.29 ± 0.17		1.37 ± 0.18		0.84 ± 0.13		1.10 ± 0.15	
0.5	1.55 ± 0.18	20	1.90 ± 0.28	39	0.98 ± 0.13	17	1.05 ± 0.16	−5
12	1.39 ± 0.25	8	1.78 ± 0.24	30	0.93 ± 0.13	11	1.42 ± 0.19	29
28	1.52 ± 0.29	18	1.79 ± 0.23	31	0.93 ± 0.14	11	1.44 ± 0.26	31

	Energy equivalents (mg O_2_ Joule^−1^)			
	Ab 30	Ab 33	Gr 30	Gr 33
Prior to thermal change	17.54	18.63	11.42	14.96
0.5	21.08	25.84	13.33	14.28
12	18.90	24.21	12.65	19.31
28	20.67	24.34	12.65	19.58

*Note*: Changes in VO_2_ (%) relative to the condition before thermal increment at each moment in time are given for comparative purposes. Energy equivalents were calculated by converting mean VO_2_ values with an oxy‐caloric coefficient of 13.6 J mg^−1^ (Lucas & Watson, 2014).

A gradual change of 4°C (Gr30) induced a metabolic response with a different temporal pattern to that induced by an abrupt change (Ab30; Figure 1b). Oxygen consumption increased to 17% of the initial VO_2_ value after 0.5 h of exposure to 30°C, but decreased to 11% and stabilised thereafter (Table 1). In addition, mean VO_2_ values in Gr30 were consistently lower than in Ab30 (Table 1; Figure 1b). A gradual change of 7°C (Gr33) also induced an increment in VO_2_, but in a lesser magnitude and with a time lapse of 12 h (Table 1; Figure 1c). Oxygen consumption in Gr33 increased in 20% of the initial value only after 12 h of sea horse exposure to 33°C and remained similar (1.44 ± 0.26 mgO_2_ h^−1^ g^−1^) at the end of the trial. Here again, VO_2_ was consistently lower in sea horses exposed to the gradual than to the abrupt thermal increment, despite the magnitude of the increment being 7°C in both cases.

### 3.2 Biochemical indicators of metabolism and antioxidant defence

**FIGURE 2:**
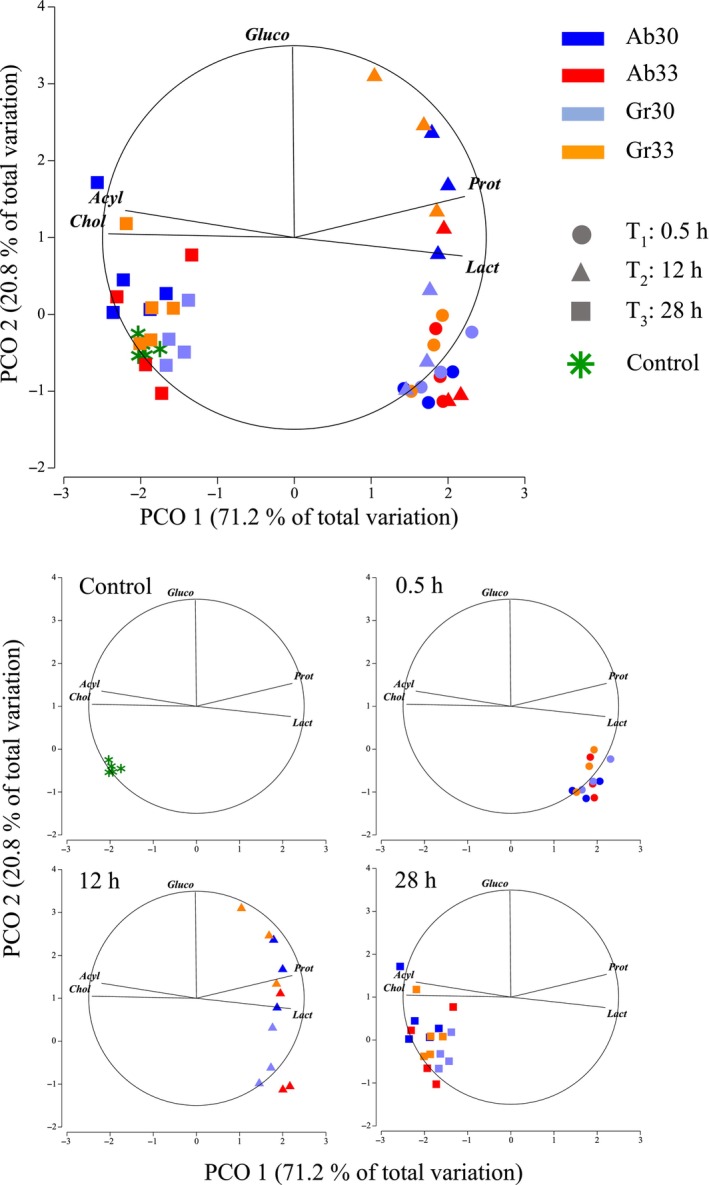
Principal coordinate ordination on metabolites (Lact, Gluco, Prot, Chol, Acyl) of juvenile *Hippocampus erectus* measured after 0.5, 12 and 28 h of exposure to abrupt and gradual increments from 26 to 30°C (4°C, Ab 30 and Gr 30) and 26 to 33°C (7°C, Ab 33 and Gr 33 respectively) and in a control group kept constant a 26°C (Control). Data were log‐transformed and normalised prior to obtaining a resemblance matric of Euclidean distances between samples.

The PCoA applied to sea horse metabolites showed that the first and second principal coordinates explained 91.9% of the total data variation (Figure 2). Ordination principally separated samples according to exposure time and, to a lesser extent, thermal treatment (Figure 2). Maps showed that samples of all thermal treatments taken after 0.5 and 12 h of exposure had high lactate and protein content but low cholesterol and acyl‐glycerides (Figure 2). These samples were clearly separated from the control group (horizontal axis, Figure 2), and differences were statistically distinguishable from random noise (Table 2).

**TABLE 2:** Results of permutational MANOVA on the metabolites (Lact, Gluco, Prot, Chol, Acyl), antioxidant enzymes and oxidative stress indicators (GSH, SOD, CAT, GST, LPO, PO) and esterase activity (AChE, CbE) of juvenile *Hippocampus erectus* after 0.5, 12 and 28 h of exposure to abrupt and gradual increments from 26 to 30°C (4°C, Ab 30 and Gr 30) and 26 to 33°C (7°C, Ab 33 and Gr 33 respectively) and in a control group kept constant a 26°C (Control).

Source	*df*	SS	MS	Pseudo‐*F*	*p*	Unique perms.
Metabolites						
Contrast C1^a^	1	22.33	22.33	4.84	<.05	9904
A: Treatment^b^	3	13.73	4.58	5.34	<.001	9944
B: Time^b^	2	151.08	75.54	88.19	<.001	9948
A × B^b^	6	16.58	2.76	3.23	<.001	9929
Residuals	34	26.28	0.77			
Total	46	230				
Antioxidant enzymes and oxidative stress indicators						
Contrast C1^a^	1	15.3	15.3	2.64	<.05	9922
A: Treatment^b^	3	22.08	7.36	1.60	.09	9923
B: Time^b^	2	57.74	28.87	6.29	<.001	9926
A × B^b^	6	28.64	4.77	1.04	.42	9914
Residuals	34	152.24	4.48			
Total	46	276				
Esterase activity						
Contrast C1^a^	1	11.76	11.76	6.59	<.01	9909
A: Treatment^b^	3	3.05	1.02	1.05	.39	9956
B: Time^b^	2	46.81	23.42	24.19	<.001	9951
A × B^b^	6	3.13	0.52	0.54	.82	9939
Residuals	34	30.15	0.89			
Total	46	92				

Abbreviations: *df*, degrees of freedom; MS, mean squares; *p*, relative cumulative frequency of pseudo‐*F*; number of unique permutations for terms in the model are given; SS, sums of squares.

^a^ First‐stage contrast between hanging control and all no‐control groups;

^b^ Second‐stage full two‐way factorial with treatment and time as fixed factors.

The significant interaction term verified that changes in the metabolite content of sea horses over time differed depending on thermal treatment (Table 2). These differences were mainly related to glucose content amongst samples taken after 12 h of exposure (vertical axis), with high values in Gr33 and Ab30 compared to low values in Gr30 and Ab33 (Figure 2). All samples taken after 28 h of exposure were close to the control group (Figure 2), indicating that metabolite concentrations in all thermal treatments had been restored to values found in sea horses that remained at 26°C constant throughout trials.

**FIGURE 3:**
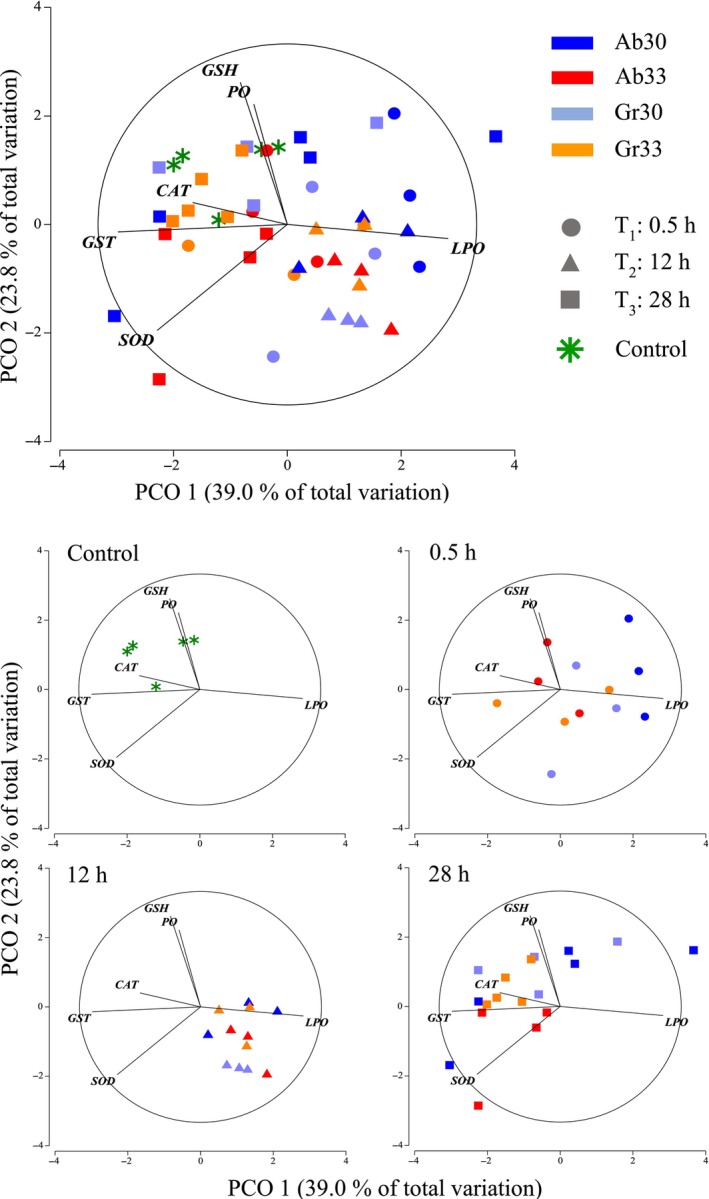
Principal Coordinate ordination on antioxidant enzymes and oxidative stress indicators (GSH, SOD, CAT, GST, LPO, PO) of juvenile *Hippocampus erectus* measured after 0.5, 12 and 28 h of exposure to abrupt and gradual increments from 26 to 30°C (4°C, Ab 30 and Gr 30) and 26 to 33°C (7°C, Ab 33 and Gr 33 respectively) and in a control group kept constant a 26°C (Control). Data were log‐transformed and normalised prior to obtaining a resemblance matric of Euclidean distances between samples.

Ordination plots of antioxidant enzymes and oxidative stress indicators in sea horses showed that the first two principal coordinates explained 62.9% of the total variation in the data (Figure 3). The first principal coordinate (horizontal axis) separated samples with high contents of LPO from those with high activity of GST, SOD and CAT. With the exception of only two sea horses from treatments Ab30 and Gr30, samples taken after 0.5 and 12 h of exposure in all thermal treatments had elevated LPO, whereas those taken after 28 h had high antioxidant activity (GST, SOD and CAT; Figure 3).

The second principal coordinate (vertical axis) separated samples with high PO and GSH and showed that the control group and samples taken after 28 h from treatments Ab30, Gr30 and Gr33 were amongst them (Figure 3). By comparison, samples from Ab33 taken at the same time showed slightly lower levels of PO and GSH. The permutational MANOVA showed a significant effect of time on antioxidant activity and oxidative stress indicators, but no statistical differences between thermal treatments could be detected (Table 2). The absence of a significant interaction term also indicated that changes through time were statistically similar for sea horses in all thermal treatments. In summary, these results showed that sea horses' antioxidant defence system responded within 0.5 h to thermal exposure; but once 28 h had elapsed levels, both enzymes and oxidative stress indicators had been re‐established to values considered normal.

**FIGURE 4:**
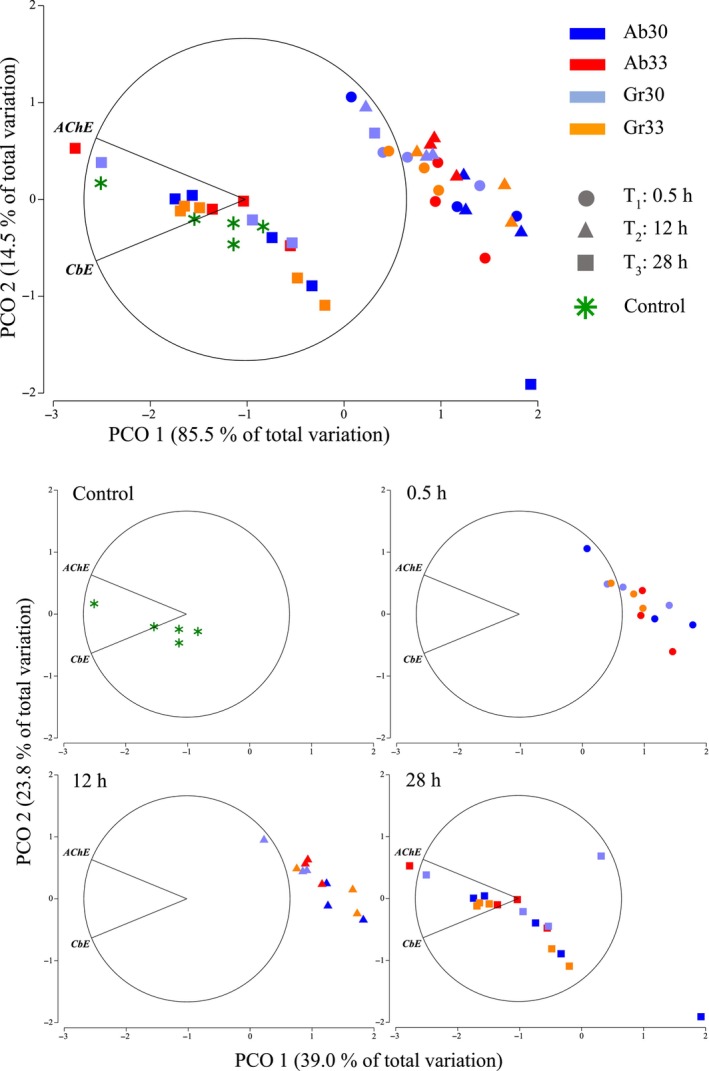
Principal Coordinate ordination on esterase activity (AChE, CbE) of juvenile *Hippocampus erectus* measured after 0.5, 12 and 28 h of exposure to abrupt and gradual increments from 26 to 30°C (4°C, Ab 30 and Gr 30) and 26 to 33°C (7°C, Ab 33 and Gr 33 respectively) and in a control group kept constant a 26°C (Control). Data were log‐transformed and normalised prior to obtaining a resemblance matric of Euclidean distances between samples.

Multivariate analysis to assess esterase activity also showed a clear separation of samples in sea horses taken after 0.5 and 12 h of thermal exposure from those taken at 28 h and a control kept constant at 26°C (Figure 4). Hypotheses testing procedures based on a permutational MANOVA found that AChE and CbE were significantly lower in juveniles *H*. *erectus* exposed to high temperatures for 0.5 and 12 h. However, normal levels of esterase activity were restored within 28 h of exposure irrespective of thermal treatment (Table 2).

## 4 DISCUSSION

The present investigation showed a distinctly consistent thermoregulatory response in juvenile *H*. *erectus* to thermal increments with magnitudes ranging from 4 to 7°C and increase rates from less than 5 min to 12 h. The main features of such response are the ability to immediately increase the *Rrout* in direct correspondence with both the magnitude and rate of thermal increase and restore metabolite concentrations, antioxidant defence, oxidative stress indicators and esterase activity to control levels within 28 h of thermal exposure. The thermoregulatory ability of *H*. *erectus* observed herein can be considered an active and successful mechanism of allostasis, since it was clearly directed to restore the homeostatic equilibrium disrupted by exposure to increasing ambient temperature (Sterling & Ever, 1988; Wendelaar Bonga, 1997). Furthermore, throughout 28 h of thermal exposure, no signs of a more permanent metabolic depression could be detected, and sea horses effectively displayed an array of physiological and biochemical mechanisms that allowed them to recover. These findings suggest that, within this time‐lapse, juvenile *H*. *erectus* have an outstanding thermal plasticity and resilience, and support the findings of previous studies that point to the capacity of sea horses to adjust to rapid temporal fluctuations in ambient temperature (Mascaró et al., 2016). However, it is important to note that, as in other species inhabiting thermally variable environments (Madeira et al., 2018; Magozzi & Calosi, 2014), these features do not protect seahorses from the deleterious effects of chronic exposure to even moderately high temperatures. Moreover, there is accumulating evidence that species with greater thermal plasticity may be at a higher risk given the high metabolic costs of their thermoregulatory response (Madeira et al., 2018; Vinagre et al., 2016). Given the steeply increasing mortality and high metabolic costs experienced by *H*. *erectus* exposed to sublethal temperatures (~30°C) for extended periods (Huipe‐Zamora, 2015), tropical populations of this species could be included in this group.

Whilst the physiological response of sea horses to increased temperature was to invariably increase *Rrout*, the magnitude and timing of the response differed depending on both magnitude and rate of thermal increase. Sea horses generally responded to temperature by elevating the *Rrout* within the first 0.5 h of exposure, but different magnitudes of thermal change produced different magnitudes in the metabolic response. An abrupt increment of 7°C (Ab33) produced a two‐fold increment in sea horse *Rrout* relative to that produced by an abrupt 4°C increment (Ab30). The direct relation between temperature and metabolic rate has been explained based on energy requirements to contend with thermal stress (Rangel & Johnson, 2018; Schulte, 2015). Oxygen is used in the tricarboxylic acid cycle and oxidative phosphorylation to produce energy through aerobic pathways. Oxidation of Acetyl‐CoA to produce CO_2_ (Akram, 2014) and reduction of coenzymes NAD+, NADP+ and FAD participate in the formation of the protonic gradient that ensures the synthesis of ATP (Kim & Gadd, 2019). Oxygen plays a part as an electron acceptor during the restoration of coenzymes to their oxidised form (Kim & Gadd, 2019). When metabolic energy is needed, the demand for oxygen at the mitochondria increases and a variety of physiological and biochemical mechanisms are triggered to assure oxygen availability at the cell (Oellermann et al., 2012). Consequently, the higher the energy demand that results from thermal stress, the larger the immediate increase in *Rrout*.

Variations in *Rrout* that followed the immediate response also differed depending on the final temperature reached in each treatment. Sea horses exposed to 30°C decreased *Rrout* from 12 h onwards, suggesting a partial compensatory effect of supplying oxygen in rates that were never higher than 18% of the initial demand. A similarly sustained exposure to 33°C, however, demanded an uninterrupted high oxygen consumption throughout 28 h, and maintained *Rrout* between 30% and 40% above the initial values. Beyond a thermal optimum, the thermoregulatory capacity of an organism depends on the time of exposure to a range of temperatures known as *pejus* (Pörtner et al., 2017; Pörtner & Knust, 2007). With increasing (or decreasing) temperature, a further thermal limit (*pessimum*) is reached where anaerobic mechanisms are activated to supply energy for cell repair and basic life maintenance (Sokolova et al., 2012). The different patterns in *Rrout* observed between seahorses exposed to 30 and 33°C irrespective of increase rate indicate that these temperatures represent different limits in a thermal continuum. Previous studies (Mascaró et al., 2016, 2019) have shown that 30°C represents a sublethal temperature at which juvenile *H*. *erectus* can acclimate and survive when exposed constantly for 30 days. However, sea horse growth rates drop to more than half compared to fish kept at optimal (25°C) and low (18°C) temperatures for the same period. Moreover, the critical thermal maxima in sea horses acclimated to 25 and 30°C was 32 and 36°C respectively (Mascaró et al., 2019). Whilst 30°C can be considered a near *pejus* temperature depending on exposure time, 33°C is a cause of more severe stress and could represent an upper thermal threshold for juvenile *H*. *erectus* from tropical populations.

Gradual thermal changes of similar magnitudes modified both the temporal pattern of the metabolic response and its magnitude. Sea horses in treatments with gradually increasing temperature showed a consistently lower *Rrout* than in their abrupt counterparts. Slower rates of thermal increase could have distributed the oxygen demand through time, allowing for partial metabolic adjustments to occur as the temperature gradually raised. In one of the first studies on the effect of temperature increase rates on fish survival, Cocking (1959) suggested that the temperature at which the common roach, *Rutilus rutilus*, died depended on the interaction between the length of exposure at lethal temperatures and the chance to acclimate at the given rate. He found that intermediate increase rates (0.4–0.8°C h ^−1^) allowed for an optimal trade‐off between partial acclimation and exposure to lethal temperatures (Cocking, 1959). No sea horse in the present study died from thermal exposure within 28 h of having reached target temperatures of 30 or 33°C, suggesting that lethal conditions were never reached. There is an indication that partial acclimation could have taken place under slow rates of thermal increase (Gr treatments), where individuals were exposed to progressively higher temperatures for longer periods than in Ab treatments. In support of this idea is that increase rates used in Gr30 and Gr33 treatments were 100 and 200 times slower than those in Ab treatments, which in turn were similar or higher than 1°C min^−1^, an increased rate used in standard methods where no thermal acclimation is expected (e.g. the dynamic method to determine critical thermal points; Lutterschmidt & Hutchison, 1997). Gradual change might have served to defer but did not avoid, the need for sufficient oxygen supply once the target temperatures had been reached and thermal exposure became constant (Table 1; Figure 1). It has been previously suggested that a gradual increase in temperature might favour the activation of biochemical and molecular mechanisms that deter cellular damage (Terblanche et al., 2007). Del Vecchio et al. (2022) found evidence that gradual thermal increments of the magnitude and rate reported herein elicited apoptotic processes involving caspase and suggested that prolonged exposure to even sublethal temperatures results in the accumulation of deleterious effects that may eventually terminate in cellular death.

Whilst the immediate increase in oxygen consumption of thermally exposed sea horses demonstrated that the main route to satisfy the energy demand is aerobic, the increased lactate concentrations registered at 0.5 and 12 h of exposure in all treatments showed anaerobiosis had been activated as part of an early thermoregulatory response. Lactate accumulates due to lactate dehydrogenase acting upon pyruvate during the anaerobic conversion of NADH to NAD+ to produce ATP (Almeida‐Val et al., 2011). In a scenario of thermal increase, theory predicts that the onset of anaerobiosis occurs when the aerobic scope becomes zero, that is, when the difference between an increasing minimum metabolic rate—in practice measured as the standard metabolic rate—and a declining maximum metabolic rate is zero (Paschke et al., 2018). Neither the minimum (or standard) nor the maximum metabolic rates of sea horses were measured in the present study, and differences in the aerobic scope could not be obtained. However, continuous registers of *Rrout* throughout experimental procedures (Appendix A) revealed a sustained, uninterrupted oxygen uptake by seahorses in all treatments as temperature increased. This suggests that the reduction in aerobic scope could have occurred as a result of a decline in the maximum metabolic rate in the first few moments of thermal exposure. Judging from changes in lactate, a thermal change of 4 to 7°C appears to have instantaneously situated sea horses in a physiological condition of a markedly reduced aerobic scope. Results suggest that before sudden exhaustion of oxygen at the mitochondria, anaerobic pathways are added to the production of urgently needed ATP. However, the low energetic efficiency of the pyruvate cycle compared to aerobic metabolic pathways (Hill et al., 2012) could have made anaerobiosis redundant under lasting conditions. This may explain why both abrupt and gradual increase to 30 and 33°C initially triggered anaerobiosis to increase ATP supply, but sea horses resumed to only aerobic pathways once the immediate high energy demand had been satisfied. Interestingly, anaerobic forms of energy supply were unnecessary within 28 h of thermal exposure, even for sea horses at temperatures near the critical thermal maxima (33°C). Future research should be directed towards a deeper understanding of the effect of hyperthermia on the different steps taking part in the mitochondrial oxidative phosphorylation and ATP production.

The availability of metabolic substrates for energy supply has important consequences in the efficacy of most regulatory mechanisms (Tseng & Hwang, 2008). Glucose circulating in the blood is the first metabolic substrate for ATP production (Polakof et al., 2011), whereas other routes to make glucose readily available may follow (Hill et al., 2012). This may explain why glucose concentrations in muscle tissues remained low after 0.5 h of exposure in all treatments and only began to increase after 12 h in Ab30 and Gr33. The temporal correspondence of these values with the high concentrations of proteins at 0.5 and 12 h further suggests that the metabolic pathway to generate glucose from proteins was effectively prompted. Protein mobilisation for energetic purposes has been previously reported in fish species as a response to ambient stress (Moon & Foster, 1995) and fasting conditions (Liang et al., 2017), as well as in response to diets with different protein, lipid and carbohydrate input (Melo et al., 2016). Within 28 h, glucose and protein concentrations in all treatments were re‐established to values similar to those in the untouched control, suggesting that overall glucose supply as a metabolic substrate (whether circulating or through gluconeogenesis) was enough to satiate the energy demand for thermoregulatory purposes. Similar results have been reported in other marine ectotherms, such as white shrimp, *Litopenaeus setiferus*, in response to a 10 day‐period of thermal stress (Pascual et al., 2003) and Atlantic salmon, *Salmo salar*, subject to swimming at 12°C and then exposed to 18°C during recovery (Galloway & Kieffer, 2003).

Temporal changes in antioxidant enzymes and oxidative stress indicators closely coupled with variations in the metabolic rate of sea horses in all thermal treatments. Given that reactive oxygen species (ROS) are responsible for oxidative stress in the cell, this connection is in strict accordance with the notion that aerobic metabolism at the mitochondria contributes to the production of 90% of all ROS (Balaban et al., 2005) and that somewhere between 0.2% and 2% of all oxygen consumed results in the ROS produced at the cell (Tirichen et al., 2021). Increased *Rrout* as a response to thermal exposure was consistently associated with high levels of LPO in samples from all treatments taken at 0.5–12 h. Lipoperoxidation compromises the stability of the cell, affecting membrane fluidity (Halliwell & Gutteridge, 1985), and its cumulative effect impairs enzyme activity, reduces the production of ATP and accelerates apoptosis (Green & Reed, 1998). In response to this toxicity, enzymes GST, CAT and SOD are the first to act as antioxidant defence (Regoli & Giuliani, 2014). SOD is considered one of the most important enzymes because of its role in converting the superoxide anion radicals to hydrogen peroxide and O_2_ (Aksnes & Njaa, 1981). Hydrogen peroxide is then reduced to water either by CAT or by glutathione peroxidase, whereas GST reduces lipid hydroperoxides to alcohol (Regoli et al., 2011). Changes in SOD, CAT and GST concentrations in sea horses of all treatments after 0.5 and 12 h of thermal exposure were consistent with these roles and provide evidence of the high sensibility of these enzymes in response to oxidative stress as part of compensatory mechanisms that balance redox equilibrium (Zhang et al., 2004). Here again, after 28 h had elapsed, levels of LPO had been restored to control values, underlining the temporary nature of the redox disequilibrium and providing further evidence of the effectiveness of the biochemical mechanisms of sea horses to control the oxidative damage derived from thermoregulation.

It is noteworthy that the relatively high levels of GSH in sea horses are maintained constantly at 26°C (control), and it suggests that the antioxidative defence is active in sea horses even under optimal thermal conditions. The cell's antioxidative defence is characterised by extremely dynamic equilibrium, sustained at a high energetic cost (Halliwell & Whiteman, 2004). GSH is the most abundant antioxidant in the cell (Gaucher et al., 2018), and plays a role either directly quenching reactive hydroxyl free radicals or as a cofactor catalysing the reduction of peroxides by glutathione peroxidase (Regoli et al., 2011). Considered the main cellular redox buffer (Gaucher et al., 2018), its high levels could provide sea horses with physiological alertness that enables rapid and effective mechanisms to respond to a temporally heterogeneous habitat. A similar strategy called ‘preparative defence’ has been previously reported in limpets of the genus *Lottia* to describe high constitutive levels of heat shock proteins as protection against periods of severe, frequent and unpredictable heat stress (Dong et al., 2008).

An esterase is a hydrolase enzyme that splits esters into an acid and an alcohol in a chemical reaction with water called hydrolysis. AChE degrades choline‐based esters, and acts in the nervous system by controlling the excitatory stimulus (Omedes et al., 2022). CbE hydrolyses chemicals containing a functional group such as a carboxylic acid ester, amide and thioester. In addition to catalysing hydrolysis, some CbE catalyses synthetic and transesterification reactions (Hosokawa & Satoh, 2002). Whilst both esterases give us information on the physiological condition of the organism, the current understanding of the effects of temperature on esterase activities is conflicting. Some studies show an increase in activity as temperature increases, while others have the opposite or no effect (Solé et al., 2015, and references therein). Results in the present study showed that both esterases decreased in activity when the thermal effect was at its highest (0.5 and 12 h after the beginning of exposure), but increased to levels similar or slightly higher than the control after 28 h. Patterns in esterase activity were, again, independent of thermal regime, further supporting that seahorse response is effective to thermal increases both abrupt and large if exposures are relatively short in duration.

When continuously exposed to a *pejus* temperature, the aerobic scope for the metabolic activity will decrease with time, compromising the correct functioning of basic biological processes such as growth, reproduction and storage (Sokolova et al., 2012). Under such circumstances, several mechanisms of acclimation in protection are triggered, which, despite their energetic cost, do not represent an immediate mortal risk (Pörtner & Knust, 2007). A previous study showed that juvenile *H*. *erectus* exposed to a thermal ramp from 25 to 30°C (1°C every 5 days) not only had similar survival rates but showed higher growth rates and thermal tolerance than control animals kept constant at 25°C during the same period. Results were explained in terms of an increased assimilation efficiency of even less ingested energy in the former compared to the latter (Mascaró et al., 2019). However, under a sustained exposure to 30°C for 30 days, sea horses showed signs of metabolic depression, grew less and increased mortality. These findings, together with those described in the current study, allow us to propose that exposure to 30°C for less than 30 days and exposures to 33°C for less than 24 h can be considered within the zone of thermal tolerance of juvenile *H*. *erectus* from Chelem Lagoon, i.e. a zone where the physiological and biochemical adaptive response is effective and individuals may recover homeostasis. Beyond these thermo‐temporal limits, the cost of satisfying the energetic demands and maintaining oxidative damage at permissible levels is unsustainable and death will eventually follow.

Because modifications to metabolism often mediate temperature‐induced effects, they disrupt species growth and reproduction and affect entire populations (Madeira et al., 2018; Pörtner et al., 2017). New climatic patterns are expected to directly influence and substantially impact nearly all species, including those aquatic (Christensen et al., 2021; Fellous et al., 2021; Larios‐Soriano et al., 2021). Under a realistic evolutionary perspective, organisms are expected to cope with ambient perturbations to their ecological niche by developing adaptive physiological traits through intra‐generational and multigenerational plasticity (Klosing et al., 2019; Román‐Palacios & Wiens, 2020; Ross et al., 2016). Understanding how physiological processes and molecular machinery in aquatic species adapt to thermal variations may help support the efforts for species conservation and improve business practices in this era of rapid climate change.

## AUTHOR CONTRIBUTIONS

**Giulia Del Vecchio:** Conceptualization (equal); data curation (equal); formal analysis (equal); investigation (equal); visualization (equal); writing – original draft (equal); writing – review and editing (equal). **Gabriela Rodríguez‐Fuentes:** Conceptualization (equal); data curation (equal); funding acquisition (equal); methodology (equal); resources (equal); writing – review and editing (equal). **Carlos Rosas:** Conceptualization (equal); data curation (equal); funding acquisition (equal); methodology (equal); resources (equal); writing – review and editing (equal). **Maite Mascaró:** Conceptualization (equal); data curation (equal); formal analysis (equal); funding acquisition (equal); investigation (equal); project administration (equal); validation (equal); writing – original draft (equal); writing – review and editing (equal).

## FUNDING INFORMATION

This work was funded by research projects PAPIIT IN‐219816, IN‐223416 (DGAPA‐UNAM). GDV received a post‐graduate grant from Consejo Nacional de Ciencia y Tecnología (CONACyT), México (CVU/Grant holder: 709829/590116).

## CONFLICT OF INTEREST STATEMENT

The authors have no relevant financial or non‐financial interests to disclose and declare that they have no conflict of interest.

## Data Availability

The datasets analysed during the current study are available in the Zenodo https://zenodo.org/record/8350051.

## APPENDIX A

### A.1

Oxygen consumption (mean values ± standard deviation) of juvenile *H*. *erectus* prior to thermal change and throughout 28 h of exposure to (a) abrupt increment from 26 to 30°C (Ab 30); (b) abrupt increment from 26 to 33°C (Ab 33); (c) gradual increment from 26 to 30°C (Gr 30) and (d) gradual increment from 26 to 33°C (Gr 33). Red dotted lines represent magnitude and rate of temperature increase in time.

## References

[ece310977-bib-0001] Akerboom, T. P. M. , & Sies, H. (1981). Assay of glutathione, glutathione disulfide, and glutathione mixed disulfides in biological samples. Methods in Enzymology, 77(C), 373–382. 10.1016/S0076-6879(81)77050-2 7329314

[ece310977-bib-0002] Akram, M. (2014). Citric acid cycle and role of its intermediates in metabolism. Cell Biochemistry and Biophysics, 68, 475–478. 10.1007/s12013-013-9750-1 24068518

[ece310977-bib-0003] Aksnes, A. , & Njaa, L. R. (1981). Catalase, glutathione peroxidase and superoxide dismutase in different fish species. Comparative Biochemistry and Physiology. B, 69, 893–896. 10.1016/0305-0491(81)90402-8

[ece310977-bib-0004] Almeida‐Val, V. M. F. , Oliveira, A. R. , Silva, M. d. N. P. d. , Ferreira‐Nozawa, M. S. , Araújo, R. M. , Val, A. L. , & Nozawa, S. R. (2011). Anoxia‐ and hypoxia‐induced expression of LDH‐A in the Amazon Oscar, *Astronotus crassipinis* . Genetics and Molecular Biology, 34, 315–322. 10.1590/S1415-47572011000200025 21734836 PMC3115329

[ece310977-bib-0005] Anderson, M. J. (2017). Permutational multivariate analysis of variance (PERMANOVA). In Wiley StatsRef: Statistics reference online. John Wiley & Sons, Ltd. 10.1002/9781118445112.stat07841

[ece310977-bib-0006] Balaban, R. S. , Nemoto, S. , & Finkel, T. (2005). Mitochondria, oxidants, and aging. Cell, 120(4), 483–495. 10.1016/j.cell.2005.02.001 15734681

[ece310977-bib-0007] Chabot, D. , Steffensen, J. F. , & Farrell, A. P. (2016). The determination of standard metabolic rate in fishes. Journal of Fish Biology, 88(1), 81–121. 10.1111/jfb.12845 26768973

[ece310977-bib-0008] Christensen, E. A. F. , Norin, T. , Tabak, I. , van Deurs, M. , & Behrens, J. W. (2021). Effects of temperature on physiological performance and behavioral thermoregulation in an invasive fish, the round goby. The Journal of Experimental Biology, 224, jeb237669. 10.1242/jeb.237669 33257434 PMC7823162

[ece310977-bib-0009] Cocking, A. W. (1959). The effects of high temperatures on roach (*Rutilus rutilus*): I. The effects of constant high temperatures. The Journal of Experimental Biology, 36, 203–216.

[ece310977-bib-0010] CONANP . (2006). Programa de Manejo de la Reserva Estatal de Dzilam, 1era. Edicion.

[ece310977-bib-0011] De la Lanza‐Espino, G. , & Gutiérrez‐Mendieta, F. J. (2017). Intervalos de parámetros no‐conservativos en sistemas acuáticos costeros de México. Hidrobiologica, 27(3), 369–390. 10.24275/uam/izt/dcbs/hidro/2017v27n3/Delalanza

[ece310977-bib-0012] Del Vecchio, G. , Galindo‐Sánchez, C. E. , Tripp‐Valdez, M. A. , López‐Landavery, E. A. , Rosas, C. , & Mascaró, M. (2022). Transcriptomic response in thermally challenged seahorses *Hippocampus erectus*: The effect of magnitude and rate of temperature change. Comparative Biochemistry and Physiology. B, 262, 110771. 10.1016/j.cbpb.2022.110771 35691555

[ece310977-bib-0013] Dong, Y. , Miller, L. P. , Sanders, J. G. , & Somero, G. N. (2008). Heat‐shock protein 70 (Hsp70) expression in four limpets of the genus Lottia: Interspecific variation in constitutive and inducible synthesis correlates with in situ exposure to heat stress. The Biological Bulletin, 215(2), 173–181. 10.2307/25470698 18840778

[ece310977-bib-0014] Ellman, G. L. , Courtney, K. D. , Andres, V. , & Featherstone, R. M. (1961). A new and rapid colorimetric determination of acetylcholinesterase activity. Biochemical Pharmacology, 7, 88–95. 10.1016/0006-2952(61)90145-9 13726518

[ece310977-bib-0015] Farrell, A. P. (2016). Pragmatic perspective on aerobic scope: Peaking, plummeting, pejus and apportioning. Journal of Fish Biology, 88, 322–343. 10.1111/jfb.12789 26592201

[ece310977-bib-0016] Fellous, A. , Wegner, K. M. , John, U. , & Mark, F. C. (2021). Windows of opportunity: Ocean warming shapes temperature‐sensitive epigenetic reprogramming and gene expression across gametogenesis and embryogenesis in marine stickleback. Global Change Biology, 28, 54–71. 10.1111/gcb.15942 34669228

[ece310977-bib-0017] Galloway, B. J. , & Kieffer, J. D. (2003). The effects of an acute temperature change on the metabolic recovery from exhaustive exercise in juvenile Atlantic Salmon (*Salmo salar*). Physiological and Biochemical Zoology, 76, 652–662. 10.1086/376921 14671713

[ece310977-bib-0018] Gaucher, C. , Boudier, A. , Bonetti, J. , Clarot, I. , Leroy, P. , & Parent, M. (2018). Glutathione: Antioxidant properties dedicated to nanotechnologies. Antioxidants, 7(5), 62. 10.3390/antiox7050062 29702624 PMC5981248

[ece310977-bib-0019] Giraud‐Billouda, M. , Rivera‐Ingrahamb, G. A. , Moreirac, D. C. , Burmesterd, T. , Castro‐Vazqueza, A. , Carvajalino‐Fernándeze, J. M. , Dafref, A. , Niug, C. , Tremblay, N. , Paitali, B. , Rosa, R. , Storeyk, M. , Vega, I. A. , Zhang, W. , Yepiz‐Plascencia, G. , Zenteno‐Savin, T. , Storey, K. , & Hermes‐Lima, M. (2019). Twenty years of the ‘preparation for oxidative stress’ (POS) theory: Ecophysiological advantages and molecular strategies. Comparative Biochemistry and Physiology Part A, 234, 36–49. 10.1016/j.cbpa.2019.1004.1004 30978470

[ece310977-bib-0020] Góth, L. (1991). A simple method for determination of serum catalase activity and revision of reference range. Clinica Chimica Acta, 196, 143–151. 10.1016/0009-8981(91)90067-M 2029780

[ece310977-bib-0021] Green, D. R. , & Reed, J. C. (1998). Mitochondria and apoptosis. Science, 281, 1309–1312. 10.1126/science.281.5381.1309 9721092

[ece310977-bib-0022] Habig, W. H. , & Jakoby, W. B. (1981). Glutathione S‐transferases (rat and human). Methods in Enzymology, 77, 218–231. 10.1016/S0076-6879(81)77029-0 6173570

[ece310977-bib-0023] Hadwan, M. H. , & Abed, H. N. (2016). Data supporting the spectrophotometric method for the estimation of catalase activity. Data in Brief, 6, 194–199. 10.1016/J.DIB.2015.12.012 26862558 PMC4707181

[ece310977-bib-0024] Halliwell, B. , & Gutteridge, J. M. C. (1985). Free radicals in biology and medicine. Journal of Free Radicals in Biology & Medicine, 1(4), 331–332. 10.1016/0748-5514(85)90140-0 3939136

[ece310977-bib-0025] Halliwell, B. , & Whiteman, M. (2004). Measuring reactive species and oxidative damage in vivo and in cell culture: How should you do it and what do the results mean? British Journal of Pharmacology, 142, 231–255. 10.1038/sj.bjp.0705776 15155533 PMC1574951

[ece310977-bib-0026] Herrera‐Silveira, J. A. , & Morales‐Ojeda, S. M. (2010). Subtropical karstic coastal lagoon assessment, Southeast Mexico the Yucatan peninsula case. In M. J. Kennisn & H. W. Paerl (Eds.), Coastal lagoons critical habitat of environmental change (pp. 307–334). CRC Press Taylor & Francis Group. 10.1017/CBO9781107415324.004

[ece310977-bib-0027] Hill, R. W. , Wyse, G. A. , & Anderson, M. (2012). Animal physiology (3rd ed.). Oxford University Press.

[ece310977-bib-0028] Hosokawa, M. , & Satoh, T. (2002). Measurement of carboxylesterase (CES) activities. Current Protocols in Toxicology, 10, 4–7. 10.1002/0471140856.TX0407S10 20945299

[ece310977-bib-0029] Huipe‐Zamora, I. V. (2015). Preferencia térmica de caballitos de mar *Hippocampus erectus*. Bachelor Degree Thesis, Universidad Michoacana de San Nicolás de Hidalgo, Mexico.

[ece310977-bib-0030] Kim, B. H. , & Gadd, G. M. (2019). Tricarboxylic acid (TCA) cycle, electron transport and oxidative phosphorylation. In Prokaryotic metabolism and physiology (pp. 80–114). Cambridge University Press. 10.1017/9781316761625.005

[ece310977-bib-0031] Kingsolver, J. G. , Higgins, J. K. , & Augustine, K. E. (2015). Fluctuating temperatures and ectotherm growth: Distinguishing non‐linear and time‐dependent effects. Journal of Experimental Biology, 218(14), 2218–2225. 10.1242/jeb.120733 25987738

[ece310977-bib-0032] Klosing, A. , Casas, E. , Hidalgo‐Carcedo, C. , Vavouri, T. , & Lehner, B. (2019). Transgenerational transmission of environmental information in *C*. *elegans* . Science, 356, 320–323. 10.1126/science.aah6412 28428426

[ece310977-bib-0033] Larios‐Soriano, E. , Re‐Araujo, A. D. , Díaz, F. , López‐Galindo, L. L. , Rosas, C. , & Ibarra‐Castro, L. (2021). Effects of recent thermal history on thermal behaviour, thermal tolerance and oxygen uptake of yellowtail kingfish (*Seriola lalandi*) juveniles. Journal of Thermal Biology, 99, 103023. 10.1016/j.jtherbio.2021.103023 34420646

[ece310977-bib-0034] Legendre, P. , & Legendre, L. (1998). Numerical ecology. In English Edition (Ed.), Developments in environmental modelling (Vol. 354, 2nd ed., pp. 1923–1939). Elsevier. 10.1098/rstb.1999.0532

[ece310977-bib-0035] Leong, C.‐M. , Tsang, T. P. N. , & Guénard, B. (2022). Testing the reliability and ecological implications of ramping rates in the measurement of critical thermal maximum. PLoS One, 17, e0265361. 10.1371/journal.pone.0265361 35286353 PMC8920270

[ece310977-bib-0036] Liang, X. , Wang, J. , Gong, G. , Xue, M. , Dong, Y. , Wu, X. , Wang, X. , Chen, C. , Liang, X. , & Qin, Y. (2017). Gluconeogenesis during starvation and refeeding phase is affected by previous dietary carbohydrates levels and a glucose stimuli during early life in Siberian sturgeon (*Acipenser baerii*). Animal Nutrition, 3, 284–294. 10.1016/j.aninu.2017.06.001 29767079 PMC5941230

[ece310977-bib-0037] Lourie, S. A. , Pollom, R. A. , & Foster, S. J. (2016). A global revision of the seahorses hippocampus Rafinesque 1810 (Actinopterygii: Syngnathiformes): Taxonomy and biogeography with recommendations for further research. Zootaxa, 4146, 1–66. 10.11646/zootaxa.4146.1.1 27515600

[ece310977-bib-0038] Lucas, A. , & Watson, J. J. (2014). Bioenergetics of aquatic animals. London CRC Press.

[ece310977-bib-0039] Lutterschmidt, W. I. , & Hutchison, V. H. (1997). The critical thermal maximum: Data to support the onset of spasms as the definitive end point. Canadian Journal of Zoology, 75, 1553–1560. 10.1139/z97-782

[ece310977-bib-0040] Madeira, C. , Mendonça, V. , Flores, A. A. V. , Diniz, M. S. , & Vinagre, C. (2018). High thermal tolerance does not protect from chronic warming – A multiple end‐point approach using a tropical gastropod, *Stramonita haemastoma* . Ecological Indicators, 91, 626–635. 10.1016/j.ecolind.2018.04.044

[ece310977-bib-0041] Magozzi, S. , & Calosi, P. (2014). Integrating metabolic performance, thermal tolerance, and plasticity enables for more accurate predictions on species vulnerability to acute and chronic effects of global warming. Global Change Biology, 21, 181–194. 10.1111/gcb.12695 25155644

[ece310977-bib-0042] Mariño‐Tapia, I. , & Enríquez, C. (2010). Estudios batimétricos y de la calidad del agua de lagunas costeras de Yucatán. Proyecto FOMIX‐CONACYT. Laboratorio de procesos costeros y oceanografía física. Mérida, Yucatán. México.

[ece310977-bib-0043] Mascaró, M. , Amaral‐Ruiz, M. , Huipe‐Zamora, I. , Martínez‐Moreno, G. , Simões, N. , & Rosas, C. (2016). Thermal tolerance and phenotypic plasticity in juvenile *Hippocampus erectus* Perry, 1810: Effect of acute and chronic exposure to contrasting temperatures. Journal of Experimental Marine Biology and Ecology, 483, 112–119. 10.1016/j.jembe.2016.07.005

[ece310977-bib-0044] Mascaró, M. , Horta, J. L. , Diaz, F. , Paschke, K. , Rosas, C. , & Simões, N. (2019). Effect of a gradually increasing temperature on the behavioural and physiological response of juvenile *Hippocampus erectus*: Thermal preference, tolerance, energy balance and growth. Journal of Thermal Biology, 85, 102406. 10.1016/j.jtherbio.2019.102406 31657747

[ece310977-bib-0045] Melo, J. F. B. , Lundstedt, L. M. , Inoue, L. A. K. , Metón, I. , Baanante, I. V. , & Moraes, G. (2016). Glycolysis and gluconeogenesis in the liver of catfish fed with different concentrations of proteins, lipids and carbohydrates. Arquivo Brasileiro de Medicina Veterinária e Zootecnia, 68, 1251–1258. 10.1590/1678-4162-8337

[ece310977-bib-0046] Mesquita, C. S. , Oliveira, R. , Bento, F. , Geraldo, D. , Rodrigues, J. V. , & Marcos, J. C. (2014). Simplified 2,4‐dinitrophenylhydrazine spectrophotometric assay for quantification of carbonyls in soxidised proteins. Analytical Biochemistry, 458, 69–71. 10.1016/J.AB.2014.04.034 24814294

[ece310977-bib-0047] Moon, T. W. , & Foster, G. D. (1995). Tissue carbohydrate metabolism, gluconeogenesis and hormonal and environmental influences. In Hochachka and Mommsen (Ed.), Biochemistry and molecular biology of fishes (pp. 65–100). Elsevier Science B.V. 10.1016/S1873-0140(06)80007-X

[ece310977-bib-0048] Oellermann, M. , Pörtner, H. O. , & Mark, F. C. (2012). Mitochondrial dynamics underlying thermal plasticity of cuttlefish (*Sepia officinalis*) hearts. The Journal of Experimental Biology, 215, 2992–3000. 10.1242/jeb.068163 22660779

[ece310977-bib-0049] Omedes, S. , Andrade, M. , Escolar, O. , Villanueva, R. , Freitas, R. , & Solé, M. (2022). B‐Esterases characterisation in the digestive tract of the common octopus and the European cuttlefish and their in vitro responses to contaminants of environmental concern. Environmental Research, 210, 112961. 10.1016/j.envres.2022.112961 35181305

[ece310977-bib-0050] Paschke, K. , Agüero, J. , Gebauer, P. , Diaz, F. , Mascaró, M. , López‐Ripoll, E. , Re, A. D. , Caamal‐Monsreal, C. , Tremblay, N. , Pörtner, H. O. , & Rosas, C. (2018). Comparison of aerobic scope for metabolic activity in aquatic ectotherms with temperature related metabolic stimulation: A novel approach for aerobic power budget. Frontiers in Physiology, 9, 01438. 10.3389/fphys.2018.01438 PMC6204536 30405425

[ece310977-bib-0051] Pascual, C. , Sánchez, A. , Sánchez, A. , Vargas‐Albores, F. , LeMoullac, G. , & Rosas, C. (2003). Haemolymph metabolic variables and immune response in *Litopenaeus setiferus* adult males: The effect of an extreme temperature. Aquaculture, 218, 637–650. 10.1016/S0044-8486(02)00300-9

[ece310977-bib-0052] Pigliucci, M. , Murren, C. J. , & Schlichting, C. D. (2006). Phenotypic plasticity and evolution by genetic assimilation. Journal of Experimental Biology, 209, 2362–2367. 10.1242/jeb.02070 16731812

[ece310977-bib-0053] Polakof, S. , Mommsen, T. P. , & Soengas, J. L. (2011). Glucosensing and glucose homeostasis: From fish to mammals. Comparative Biochemistry and Physiology Part B: Biochemistry and Molecular Biology, 160, 123–149. 10.1016/j.cbpb.2011.07.006 21871969

[ece310977-bib-0054] Poloczanska, E. S. , Burrows, M. T. , Brown, C. J. , García Molinos, J. , Halpern, B. S. , HoeghGuldberg, O. , Kappel, C. V. , Moore, P. J. , Richardson, A. J. , Schoeman, D. S. , & Sydeman, W. J. (2016). Responses of marine organisms to climate change across oceans. Frontiers in Marine Science, 3, 1–21. 10.3389/fmars.2016.00062

[ece310977-bib-0055] Pörtner, H. O. (2002). Climate variations and the physiological basis of temperature dependent biogeography: Systemic to molecular hierarchy of thermal tolerance in animals. Comparative Biochemistry and Physiology. Part A, Molecular & Integrative Physiology, 132, 739–761. 10.1016/S1095-6433(02)00045-4 12095860

[ece310977-bib-0056] Pörtner, H. O. , Bock, C. , & Mark, F. C. (2017). Oxygen‐ and capacity‐limited thermal tolerance: Bridging ecology and physiology. The Journal of Experimental Biology, 220, 2685–2696. 10.1242/jeb.134585 28768746

[ece310977-bib-0057] Pörtner, H. O. , & Knust, R. (2007). Climate change affects marine fishes through the oxygen limitation of thermal tolerance. Science, 315, 95–98. 10.1126/science.1135471 17204649

[ece310977-bib-0058] Rahaman, M. S. , & Rahaman, M. S. (2021). Effects of elevated temperature on prooxidant‐antioxidant homeostasis and redox status in the American oyster: Signaling pathways of cellular apoptosis during heat stress. Environmental Research, 196, 110428. 10.1016/j.envres.2020.110428 33186574

[ece310977-bib-0059] Rangel, R. E. , & Johnson, D. W. (2018). Metabolic responses to temperature in a sedentary reef fish, the bluebanded goby (*Lythrypnus dalli*, Gilbert). Journal of Experimental Marine Biology and Ecology, 501, 83–89. 10.1016/j.jembe.2018.01.011

[ece310977-bib-0060] Regoli, F. , & Giuliani, M. E. (2014). Oxidative pathways of chemical toxicity and oxidative stress biomarkers in marine organisms. Marine Environmental Research, 93, 106–117. 10.1016/j.marenvres.2013.07.006 23942183

[ece310977-bib-0061] Regoli, F. , Giuliani, M. E. , Benedetti, M. , & Arukwe, A. (2011). Molecular and biochemical biomarkers in environmental monitoring: A comparison of biotransformation and antioxidant defense systems in multiple tissues. Aquatic Toxicology, 1055, 56–66. 10.1016/j.aquatox.2011.06.014 22099345

[ece310977-bib-0062] Rodríguez‐Fuentes, G. , Armstrong, J. , & Schlenk, D. (2008). Characterisation of muscle cholinesterases from two demersal flatfish collected near a municipal wastewater outfall in Southern California. Ecotoxicology and Environmental Safety, 69, 466–471. 10.1016/J.ECOENV.2007.06.008 17659776

[ece310977-bib-0063] Roessig, J. M. , Woodley, C. M. , Cech, J. J. , & Hansen, L. J. (2004). Effects of global climate change on marine and estuarine fishes and fisheries. Reviews in Fish Biology and Fisheries, 14, 251–275. 10.1007/s11160-004-6749-0

[ece310977-bib-0064] Román‐Palacios, C. , & Wiens, J. J. (2020). Recent responses to climate change reveal the drivers of species extinction and survival. Proceedings of the National Academy of Sciences of the United States of America, 117, 4211–4217. 10.1073/pnas.1913007117 32041877 PMC7049143

[ece310977-bib-0065] Ross, P. M. , Parker, L. P. , & Byrne, M. (2016). Transgenerational responses of molluscs and echinoderms to changing ocean conditions. ICES Journal of Marine Science, 73, 537–549. 10.1093/icesjms/fsv254

[ece310977-bib-0066] Schulte, P. M. (2015). The effects of temperature on aerobic metabolism: Towards a mechanistic understanding of the responses of ectotherms to a changing environment. Journal of Experimental Biology, 218, 1856–1866. 10.1242/jeb.118851 26085663

[ece310977-bib-0067] Sokolova, I. M. , Frederich, M. , Bagwe, R. , Lannig, G. , & Sukhotin, A. A. (2012). Energy homeostasis as an integrative tool for assessing limits of environmental stress tolerance in aquatic invertebrates. Marine Environmental Research, 79, 1–15. 10.1016/j.marenvres.2012.04.003 22622075

[ece310977-bib-0068] Solé, M. , Varó, I. , González‐Mira, A. , & Torreblanca, A. (2015). Xenobiotic metabolism modulation after long‐term temperature acclimation in juveniles of *Solea senegalensis* . Marine Biology, 162(2), 401–412. 10.1007/s00227-014-2588-2

[ece310977-bib-0069] Somero, G. N. (2015). Temporal patterning of thermal acclimation: From behavior to membrane biophysics. The Journal of Experimental Biology, 218, 167–169. 10.1242/jeb.109843 25609779

[ece310977-bib-0070] Sterling, P. , & Ever, J. (1988). Allostasis: A new paradigm to explain arousal pathology. In S. Fisher & J. Reason (Eds.), Handbook of life stress, cognition and health (Vol. 28, pp. 629–649). Wiley. 10.1016/0005-7967(90)90076-U

[ece310977-bib-0071] Tepolt, C. K. , & Somero, G. N. (2014). Master of all trades: Thermal acclimation and adaptation of cardiac function in a broadly distributed marine invasive species, the European green crab, *Carcinus maenas* . Journal of Experimental Biology, 217(7), 1129–1138. 10.1242/JEB.093849 24671964

[ece310977-bib-0072] Terblanche, J. S. , Deere, A. J. , Clusella‐Trullas, S. , Janion, C. , & Chown, S. L. (2007). Critical thermal limits depend on methodological context. Proceedings of the Royal Society B, 274, 2935–2942. 17878142 10.1098/rspb.2007.0985 PMC2291155

[ece310977-bib-0073] Tirichen, H. , Yaigoub, H. , Xu, W. , Wu, C. , Li, R. , & Li, Y. (2021). Mitochondrial reactive oxygen species and their contribution in chronic kidney disease progression through oxidative stress. Frontiers in Physiology, 12, 627837. 10.3389/fphys.2021.627837 33967820 PMC8103168

[ece310977-bib-0074] Tseng, Y.‐C. , & Hwang, P.‐P. (2008). Some insights into energy metabolism for osmoregulation in fish. Comparative Biochemistry and Physiology Part C: Toxicology & Pharmacology, 148, 419–429. 10.1016/j.cbpc.2008.04.009 18539088

[ece310977-bib-0075] Vinagre, C. , Leal, I. , Mendonça, V. , Madeira, D. , Narciso, L. , Diniz, M. S. , & Flores, A. A. V. (2016). Vulnerability to climate warming and acclimation capacity of tropical and temperate coastal organisms. Ecological Indicators, 62, 317–327. 10.1016/j.ecolind.2015.11.010

[ece310977-bib-0076] Wendelaar Bonga, S. E. (1997). The stress response in fish. Physiological Reviews, 77(3), 591–625. http://physrev.physiology.org/cgi/content/abstract/77/3/591 9234959 10.1152/physrev.1997.77.3.591

[ece310977-bib-0077] Wernberg, T. , Russell, B. D. , Moore, P. J. , Ling, S. D. , Smale, D. A. , Campbell, A. , Coleman, M. A. , Steinberg, P. D. , Kendrick, G. A. , & Connell, S. D. (2011). Impacts of climate change in a global hotspot for temperate marine biodiversity and ocean warming. Journal of Experimental Marine Biology and Ecology, 400, 7–16. 10.1016/j.jembe.2011.02.021

[ece310977-bib-0078] Zhang, J. , Shen, H. , Wang, X. , Wu, J. , & Xue, Y. (2004). Effects of chronic exposure of 2,4‐dichlorophenol on the antioxidant system in liver of freshwater fish *Carassius auratus* . Chemosphere, 55, 167–174. 10.1016/j.chemosphere.2003.10.048 14761689

